# Exploration of the Long Noncoding RNAs Involved in the Crosstalk between M2 Macrophages and Tumor Metabolism in Lung Cancer

**DOI:** 10.1155/2023/4512820

**Published:** 2023-01-25

**Authors:** Fang Fang, Yuanshan Yao, Zhe Ma

**Affiliations:** ^1^Department of Medical Ultrasound, The First Affiliated Hospital of Shandong First Medical University & Shandong Provincial Qianfoshan Hospital, Shandong Medicine and Health Key Laboratory of Abdominal Medical Imaging, Jinan, Shandong, China; ^2^Department of Thoracic Surgery, Shanghai Key Laboratory of Clinical Geriatric Medicine, Huadong Hospital Affiliated to Fudan University, Shanghai, China

## Abstract

**Background:**

Complex regulation exists between tumor metabolism and M2 macrophages. Long noncoding RNAs (lncRNAs) are famous for their wide regulatory role. This study aimed to identify the lncRNAs involved in the crosstalk between tumor metabolism and M2 macrophages.

**Methods:**

The Cancer Genome Atlas was responsible for the public data. R software was responsible for the analysis of public data.

**Results:**

Based on the input expression profile, we quantified the M2 macrophage infiltration using the CIBERSORT algorithm and found that M2 macrophages were a risk factor for lung cancer. Also, we found that M2 macrophages were correlated with multiple metabolism pathways. Then, 67 lncRNAs involved in both M2 macrophages and related metabolism pathways were identified. A prognosis signature based on AC027288.3, AP001189.3, FAM30A, GAPLINC, LINC00578, and LINC01936 was established, which had good prognosis prediction ability. The clinical parameters and risk score were combined into a nomogram plot for better prediction of the patient's prognosis. A high fit of actual survival and nomogram-predicted survival was found using the calibration plot. Moreover, in low-risk patients, immunotherapy was more effective, while cisplatin and docetaxel were more effective in high-risk patients. Biological enrichment analysis indicated pathways of notch signaling, TGF-*β* signaling, interferon alpha response, and interferon-gamma response were activated in the high-risk group. Meanwhile, the risk score was associated with tumor metabolism and M2 macrophages. Also, we found that the promoting effect of CAPLINC on M2 macrophage polarization might act through multiple metabolism pathways.

**Conclusions:**

Our result can provide new insights into the interaction between M2 macrophages and tumor metabolism, as well as the involved lncRNAs, which can provide the direction for future studies.

## 1. Introduction

As the most commonly diagnosed malignancy, lung cancer has a high medical burden [1]. Annually, lung cancer can lead to nearly 1.5 million new cases, and the number is rising relentlessly [2]. Surgical treatment for lung cancer in the early stages can effectively improve long-term survival. Nevertheless, it is common for patients to have delayed diagnosis due to hidden clinical symptoms, which can directly result in treatment delay and poor survival [2]. It is, therefore, crucial to identify possible diagnostic and therapeutic targets for lung cancer.

In the tumor microenvironment (TME), macrophages play a key role [3]. In tumorigenesis, macrophages contribute to the formation of an inflammatory microenvironment, leading to high mutational characteristics in cells. Also, as tumors progress, macrophages can stimulate tumor cell migration and invasion, inhibit antitumor immunity, and stimulate angiogenesis [4]. In general, functional M2 macrophages are associated with tumor promotion and have attracted much attention [4]. In lung cancer, Lu et al. revealed that the overexpressed Oct4 directly increases the level of M-CSF in A549 cells, contributing to M2 macrophage polarization and tumor progression [5]. Immunity and metabolism are closely linked in the tumor microenvironment. For instance, Palsson-McDermott et al. found that under the induction of LPS, PKM2 (pyruvate kinase M2), a metabolic regulator, can form a complex with Hif-1*α*, thereby regulating glycolytic reprogramming and succinate production, which is a key determinant of macrophage activation [6]. Additionally, Hinshaw et al. indicated that Hedgehog signaling can significantly affect the metabolic processes of macrophages, like lipid metabolism, mitochondrial adaptations, and metabolic sensing [7]. Specifically, a reduction in the flux through the UDP-GlcNAc biosynthesis pathway was observed when Hedgehog signaling was inhibited in M2 macrophages [7]. Long noncoding RNAs (lncRNAs) are a class of noncoding RNA molecules longer than 200 nucleotides, which can greatly influence tumor development [8]. Cao et al. found that the lncRNA MM2P could be the modulator of macrophage M2 polarization [9]. Research has also shown that lncRNAs have a wide range of regulatory effects on tumor metabolism [10]. Consequently, exploring the lncRNAs that could affect tumor metabolism and M2 macrophages may provide novel insights for lung cancer therapy options.

Secondary analyses of big data and public studies have enhanced researchers' understanding of diseases since the age of big data arrived [11–13]. Based on the convenience brought by the open-access data, we systematically explored the underlying interactions between M2 macrophages, tumor metabolism, and lncRNAs. Also, a prognosis model based on AC027288.3, AP001189.3, FAM30A, GAPLINC, LINC00578, and LINC01936 was established. The prognosis model showed satisfactory performance in predicting patient survival. Also, patients in different risk groups might have different response performances on immunotherapy and chemotherapy. GAPLINC was selected for further analysis. Our results improve the metabolism regulatory network of M2 macrophages, as well as the role of GAPLINC in cancers, especially lung cancer.

## 2. Methods

### 2.1. Public Data Extraction

The public second sequence file and clinical parameters of non-small cell lung cancer were obtained from The Cancer Genome Atlas (TCGA) database, LUAD, and LUSC projects. The original files of the second sequence information are in “STAR-Counts” form, and clinical parameters are in “BCR-XML” form. The human reference genomic file GRCh38.p13 version obtained from the Ensembl website was utilized for probe annotation. All the data were sorted out using the R code. From the TCGA database, microsatellite instability (MSI) and tumor mutational burden (TMB) were obtained. The score of mRNAsi and EREG-mRNAsi of each lung cancer patient was obtained from the previous study [14]. The baseline information of involved patients is shown in Table 1.

### 2.2. Immune Cell Quantification

Using the CIBERSORT algorithm, 22 immune cells were quantified in TME, including M2 macrophages [15]. CIBERSORT is a useful method for the high-throughput characterization of different cell types (such as TILs) in complex tissues. The detailed gene expression profile of each patient was in the input file.

### 2.3. Biological Enrichment and Metabolism Quantification

Underlying biological differences were identified using the gene set enrichment analysis (GSEA) [16]. The hallmark pathway set was selected as the reference enrichment file. The single-sample GSEA (ssGSEA) algorithm was utilized to quantify the normalized enrichment score of metabolism terms, whose reference file wasKEGG.v7.5.1.GMT, which was downloaded from the MSIGDB website. Gene Ontology (GO) analysis was conducted using the clusterProfiler package [17].

### 2.4. Prognosis Performance and Evaluation

For the selected genes or other continuous variables, univariate Cox regression was utilized to identify prognosis-related variables (*P* < 0.05). LASSO regression was utilized to select optimization variables through dimensionality reduction. For the variables screened by LASSO regression, multivariate Cox regression was used to establish a signature for prognosis prediction, whose formula is “risk score = expression of variable A ^*∗*^ coef A + expression of variable B ^*∗*^ coef B + … + expression of variable N ^*∗*^ Coef N”. Kaplan–Meier (KM) survival curve was utilized to compare the survival differences between different groups. The receiver operating characteristic (ROC) curve was used to assess the prediction performance of variables on specific outcomes. A nomogram plot was utilized to combine clinical parameters and risk score for better clinical practice. Calibration curves were utilized to calculate the fit between actual outcomes and the nomogram-predicted survival. Decision curve analysis (DCA) was utilized to assess the performance of the nomogram plot. For the prognosis model establishment, randomization was used to assign patients to the training and validation groups with a 1 : 1 ratio.

### 2.5. Sensitivity of Immunotherapy and Chemotherapy

The evaluation of immunotherapy was conducted using the Tumor Immune Dysfunction and Exclusion (TIDE) analysis [18]. In the TIDE analysis, the “cancer type” was selected as “NSCLC.” Through TIDE analysis, each patient was assigned a TIDE score based on their gene expression information, in which a score <0 was defined as an immunotherapy responder; otherwise, a nonresponders. The sensitivity of patients to chemotherapy was evaluated by the Genomics of Drug Sensitivity in Cancer (GDSC) database [19].

### 2.6. Statistical Analysis

For the public data, R software was utilized for algorithm operation, statistical analysis, and plotting. GraphPrism was used for the data generated by the experiments. *P* value less than 0.05 was regarded as statistically significant.

## 3. Results

### 3.1. M2 Macrophages in Lung Cancer

**Figure 1:**
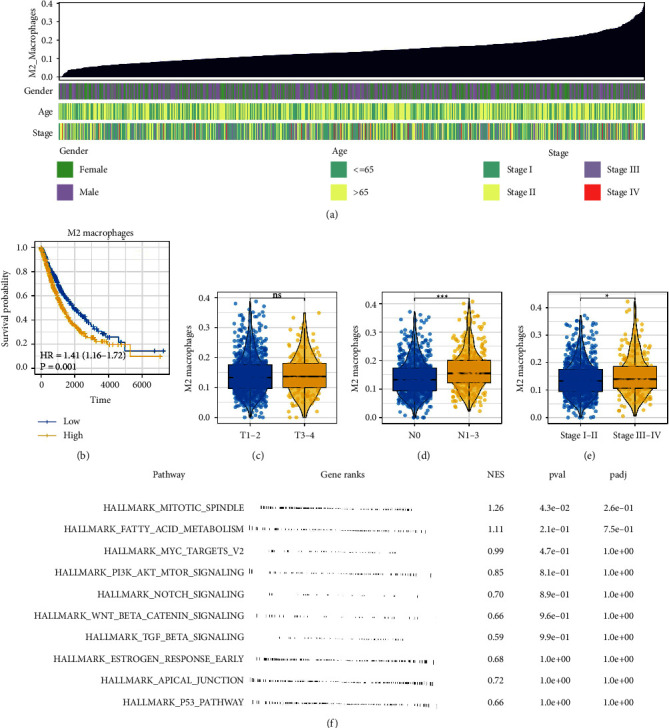
Exploration of M2 macrophages in lung cancer. (a) Quantification of M2 macrophages through the CIBERSORT algorithm in lung cancer; (b) KM survival curves of patients with high and low M2 macrophage infiltration; (c)–(e) M2 macrophage infiltration difference in patients with different clinical features, including T stage, N stage, and clinical stage, ns =*P* > 0.05, ^*∗*^=*P* < 0.05, ^*∗∗∗*^=*P* < 0.001; (f) biological enrichment of M2 macrophages based on the hallmark gene set.

The whole flowchart of this study is shown in Figure S1. Based on the input expression profile, we quantified the M2 macrophage infiltration using the CIBERSORT algorithm (Figure 1(a)). KM survival curves indicated that patients with higher M2 macrophages had a poor prognosis (Figure 1(b)). Furthermore, we compared the M2 macrophage difference in patients with different clinical parameters. Results indicated M2 macrophages might be associated with worse N and clinical stages, but not T stages (Figures 1(c)–1(e)). Biological enrichment based on the hallmark set showed that pathways of the mitotic spindle and fatty acid metabolism were activated in the patients with high M2 macrophage levels (Figure 1(f)).

### 3.2. M2 Macrophages Are Correlated with Multiple Metabolism Pathways

**Figure 2:**
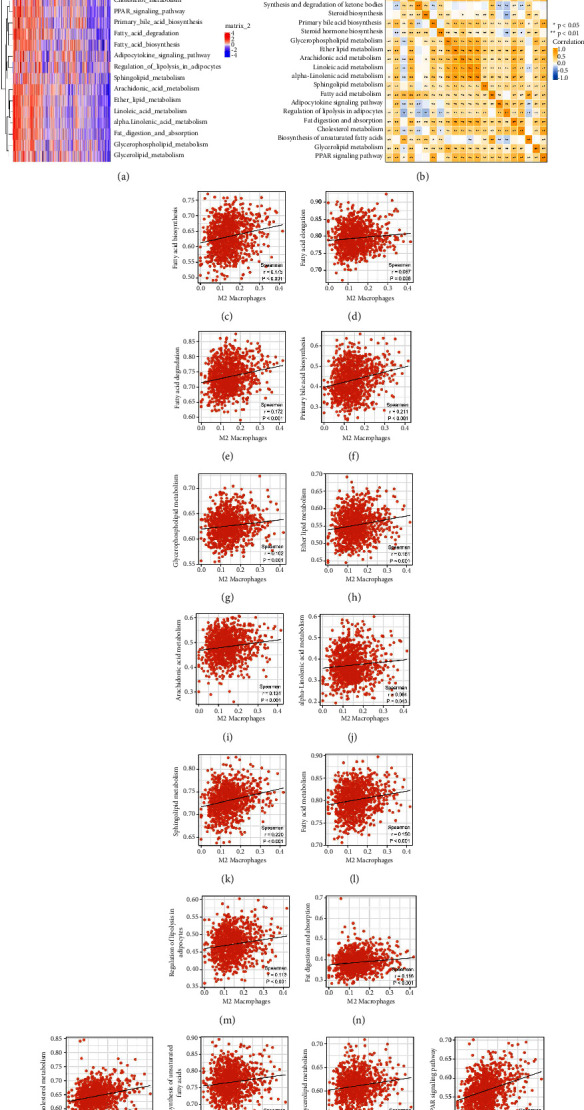
Correlation between M2 macrophages and metabolism. (a) The ssGSEA algorithm was used to quantify the tumor metabolism status; (b) the coregulation relationship between these metabolic pathways and M2 macrophages; (c)–(r) the correlation of quantified metabolism terms with M2 macrophages.

Then, we tried to explore the potential interaction between M2 macrophages and tumor metabolism. The 21 common metabolism pathways were quantified using the ssGSEA analysis (Figure 2(a)).  Figure 2(b) presents the coregulation relationship between these metabolic pathways and M2 macrophages. A positive correlation was found between M2 macrophages and fatty acid biosynthesis (Figure 2(c), correlation = 0.175, *P* < 0.001), fatty acid elongation (Figure 2(d), correlation = 0.087, *P*=0.006), fatty acid degradation (Figure 2(e), correlation = 0.172, *P* < 0.001), primary bile acid biosynthesis (Figure 2(f), correlation = 0.211, *P* < 0.001), glycerophospholipid metabolism (Figure 2(g), correlation = 0.102, *P*=0.001), ether lipid metabolism (Figure 2(h), correlation = 0.161, *P* < 0.001), arachidonic acid metabolism (Figure 2(i), correlation = 0.131, *P* < 0.001), alpha-linolenic acid metabolism (Figure 2(j), correlation = 0.064, *P*=0.043), sphingolipid metabolism (Figure 2(k), correlation = 0.220, *P* < 0.001), fatty acid metabolism (Figure 2(l), correlation = 0.150, *P* < 0.001), regulation of lipolysis in adipocytes (Figure 2(m), correlation = 0.113, *P* < 0.001), fat digestion and absorption (Figure 2(n), correlation = 0.116, *P* < 0.001), cholesterol metabolism (Figure 2(o), correlation = 0.241, *P* < 0.001), biosynthesis of unsaturated fatty acids (Figure 2(p), correlation = 0.122, *P* < 0.001), glycerolipid metabolism (Figure 2(q), correlation = 0.138, *P* < 0.001), and PPAR signaling pathway (Figure 2(r), correlation = 0.292, *P* < 0.001).

### 3.3. Establishment of the Prognosis Model

**Figure 3:**
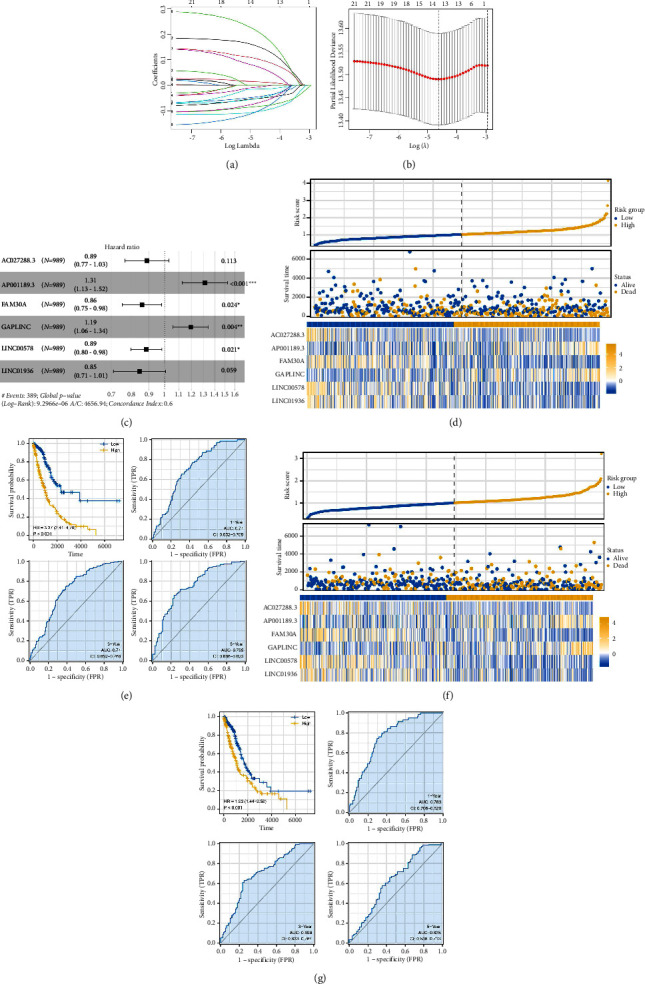
Construction and validation of prognosis signature. (a and b) LASSO regression of input genes; (c) multivariate Cox regression based on the genes identified by LASSO regression; (d) overview of the risk score in the training cohort; (e) KM survival and ROC curve of high- and low-risk patients in the training cohort; (f) overview of the risk score in the validation cohort; (g) KM survival and ROC curve of high- and low-risk patients in the validation cohort.

We have found that M2 macrophages are correlated with multiple metabolism pathways. Following this, the genes involved in these metabolism pathways were screened. Then, the lncRNAs meeting the criteria of |Cor > 0.3| and *P* < 0.05 were identified, which might be involved in the crosstalk of tumor metabolism and M2 macrophages. Among these lncRNAs, 67 lncRNAs were remarkably correlated with M2 macrophages; therefore, they are selected for the following analysis (Figure S2). LncRNAs that were significantly related to patients' survival with *P* < 0.05 were screened using the univariate Cox regression. LASSO regression was utilized to identify the optimal variable (Figures 3(a) and 3(b)). Multivariate Cox regression selected six lncRNAs used for prognosis signature establishment, consisting of AC027288.3, AP001189.3, FAM30A, GAPLINC, LINC00578, and LINC01936 (risk score = AC027288.3 ^*∗*^ −0.118 + AP001189.3 ^*∗*^ 0.269 + FAM30A ^*∗*^ −0.151 + GAPLINC ^*∗*^ 0.175 + LINC00578 ^*∗*^ −0.122 + LINC01936 ^*∗*^ −0.168) (Figure 3(c)). In the training cohort, high-risk patients were more likely to be dead cases (Figure 3(d)). KM survival curve indicated that high-risk patients had a worse survival than low-risk patients (Figure 3(e), HR = 3.37, *P* < 0.001). Moreover, the ROC curve showed a good prediction ability of the risk score on patient's prognosis (Figure 3(e), 1-, 3-, and 5-year AUC = 0.71, 0.71, and 0.755, respectively). Results in the validation cohort show the same trend (Figure 3(f)), as well as satisfactory prediction performance (Figure 3(g), HR = 1.93, *P* < 0.001; 1-, 3- and 5-year AUC = 0.71, 0.71, and 0.755, respectively).

### 3.4. Clinical Correlation and Nomogram

**Figure 4:**
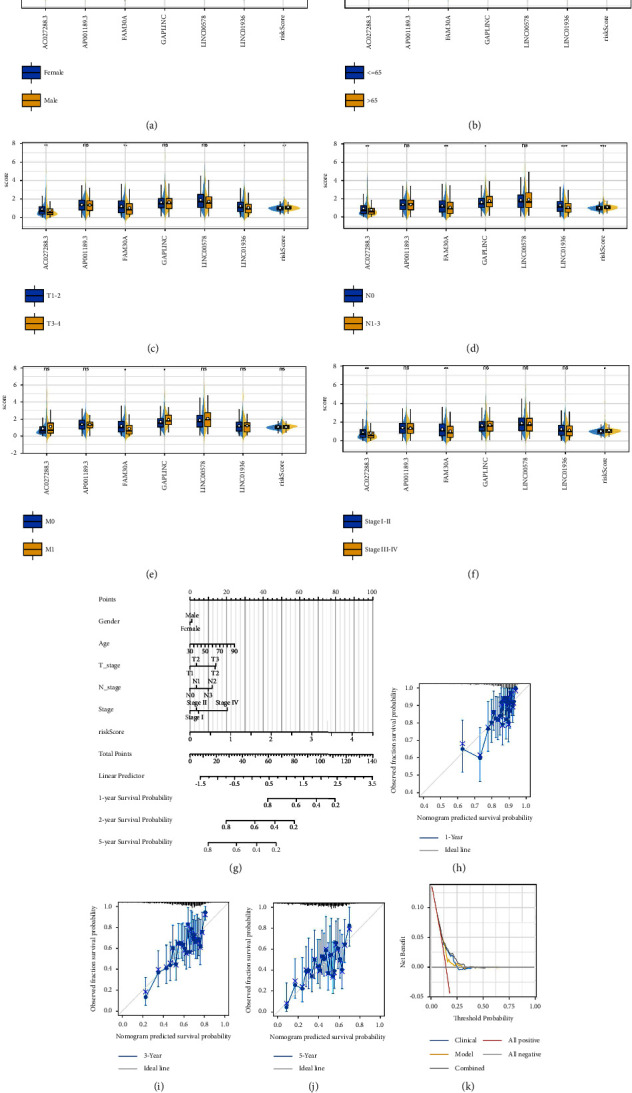
Clinical correlation analysis and nomogram. (a)–(f) The level of model lncRNAs and risk score in patients with different clinical features, ns =*P* > 0.05, ^*∗*^=*P* < 0.05, ^*∗∗*^=*P* < 0.01, ^*∗∗∗*^=*P* < 0.001; (g) a nomogram plot combining risk score and clinical features; (h)–(j) the 1, 3, and 5 years calibration curves of nomogram; (k) DCA curve of the nomogram.

The difference in clinical parameters can directly lead to diverse prognosis outcomes. Consequently, we investigated the level of risk score and model lncRNAs in patients with different clinical features. Results showed that AC027288.3, FAM30A, LINC00578, and LINC01936 were upregulated in the female patients, while risk score was upregulated in male patients (Figure 4(a)); LINC00578 was upregulated in patients <65 years old, while LINC01936 was upregulated in the patients >65 years old (Figure 4(b)); AC02788.3, FAM30A, and LINC01936 were upregulated in the T1-2 patients, while risk score was upregulated in the T3-4 patients (Figure 4(c)); AC02788.3, FAM30A, and LINC01936 were upregulated in the N0 patients, while GAPLINC and risk score were upregulated in the N1-3 patients (Figure 4(d)); FAM30A was upregulated in the M0 patients, while GAPLINC was upregulated in the M1 patients (Figure 4(e)); AC02788.3 and FAM30A were upregulated in the stage I-II patients, while risk score was upregulated in the stage III-IV patients (Figure 4(f)). Next, the clinical parameters and risk score were combined into a nomogram plot for better prediction performance of patients' prognosis (Figure 4(g)). A high fit between actual survival and nomogram-predicted survival was found using the calibration plot (Figures 4(h)–4(j)). The DCA curve showed that the nomogram had a better performance than both risk score and clinical parameters (Figure 4(k)). Univariate and multivariate analyses showed that our model is a risk factor independent of other clinical parameters (Figures S3A and S3B).

### 3.5. High- and Low-Risk Patients Have Different Sensitivities to Immunotherapy and Chemotherapy

**Figure 5:**
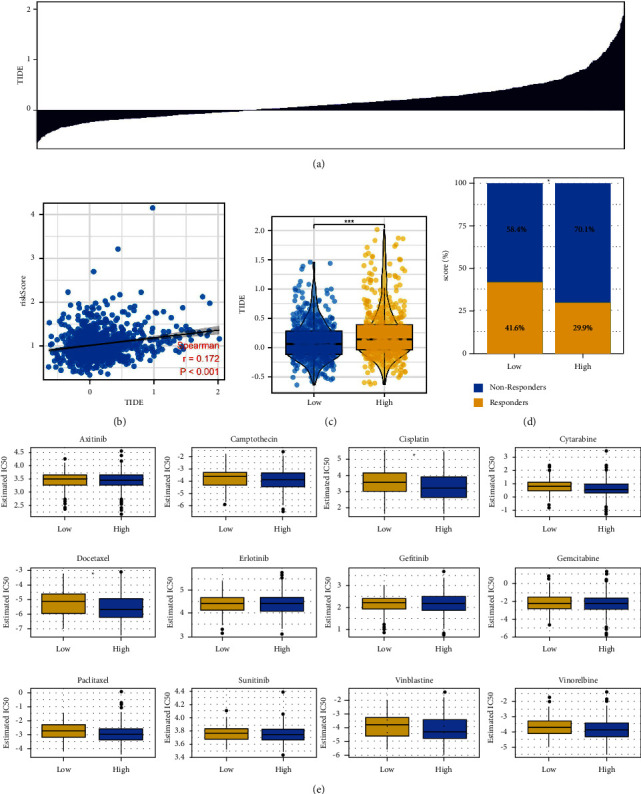
Immunotherapy and chemotherapy sensitivity. (a) The TIDE algorithm was used to evaluate the response of lung cancer patients on immunotherapy; (b) correlation between risk score and TIDE score; (c and d) low-risk patients had a lower TIDE score and a high percentage of immunotherapy responders, ^*∗*^=*P* < 0.05; (e) chemotherapy sensitivity difference between high- and low-risk patients, ^*∗*^=*P* < 0.05.

The TIDE algorithm was used to calculate the TIDE score of each patient, which can reflect the patient's response to immunotherapy (Figure 5(a)). A positive correlation was found between the risk score and the TIDE score (Figure 5(b), correlation = 0.172, *P* < 0.001). According to the results, low-risk patients had a lower TIDE score and a higher proportion of patients who responded to immunotherapy (Figures 5(c) and 5(d), 41.6% vs. 29.9%). Additionally, high-risk patients were more sensitive to cisplatin and docetaxel (Figure 5(e)).

### 3.6. Risk Score Was Associated with Tumor Metabolism and M2 Macrophages

**Figure 6:**
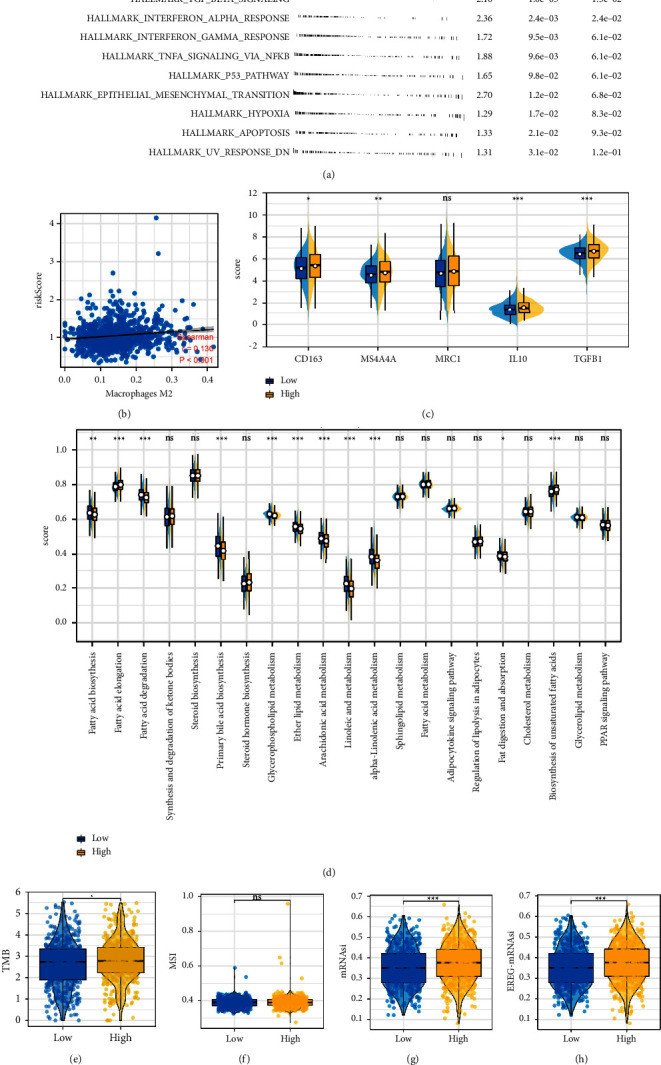
Metabolism and M2 macrophage infiltration differences in patients with different risk groups. (a) Biological enrichment of the risk score based on the hallmark gene set; (b) correlation between the risk score and M2 macrophage infiltration; (c) M2 macrophage-related molecules were highly expressed in the high-risk patients; (d) metabolism difference between high- and low-risk patients; (e)–(h) TMB, MSI, mRNAsi, and EREG-mRNAsi difference between high- and low-risk patients.

Biological enrichment analysis indicated that pathways of notch signaling, TGF-*β* signaling, interferon alpha response, and interferon-gamma response were activated in the high-risk group (Figure 6(a)). Meanwhile, we found a significant correlation between risk score and M2 macrophages (Figure 6(b), correlation = 0.130, *P* < 0.001). In addition, M2 macrophage-related molecules were highly expressed in the high-risk patients (Figure 6(c)). Furthermore, high-risk patients might have higher fatty acid elongation and biosynthesis of unsaturated fatty acids but lower fatty acid biosynthesis, fatty acid degradation, primary bile acid biosynthesis, glycerophospholipid metabolism, ether lipid metabolism, arachidonic acid metabolism, and linoleic acid metabolism levels (Figure 6(d)). A higher TMB, mRNAsi, and EREG-mRNAsi level were observed in the high-risk patients, but not MSI (Figures 6(e)–6(h)). Next, we explored the biological role of identified model lncRNAs, including AC027288.3, AP001189.3, FAM30A, GAPLINC, LINC00578, and LINC01936. Results showed that for the patients with high AC027288.3 expression, the pathways of myogenesis, allograft rejection, epithelial-mesenchymal transition, coagulation, and angiogenesis were enriched in (Figure S4A); for the patients with high AP001189.3 expression, the pathways of angiogenesis, TGF-*β* signaling, IL6/JAK/STAT3 signaling, coagulation, complement, epithelial-mesenchymal transition, and apical junction were enriched in (Figure S4B); for the patients with high FAM30A expression, the pathways of allograft rejection, interferon-gamma response, IL6/JAK/STAT3 signaling, interferon alpha response, inflammatory response, and IL2/STAT5 signaling were enriched in (Figure S4C); for the patients with high GAPLINC expression, the pathways of angiogenesis, TGF-*β* signaling, IL6/JAK/STAT3 signaling, interferon alpha response, protein secretion, and bile acid metabolism were enriched in (Figure S4D); for the patients with high LINC00578 expression, the pathways of epithelial-mesenchymal transition, inflammatory response, allograft rejection, angiogenesis, and IL6/JAK/STAT3 signaling were enriched in (Figure S4E); for the patients with high LINC01936 expression, the pathways of allograft rejection, interferon gamma response, myogenesis, inflammatory response, KRAS signaling, and epithelial-mesenchymal transition were enriched in (Figure S4F). Moreover, results indicated that risk score was positively correlated with the M0 macrophages, not the M1 macrophages (Figures S5A and S5B).

### 3.7. GAPLINC May Affect M2 Macrophages Polarization through Multiple Metabolism Pathways

**Figure 7:**
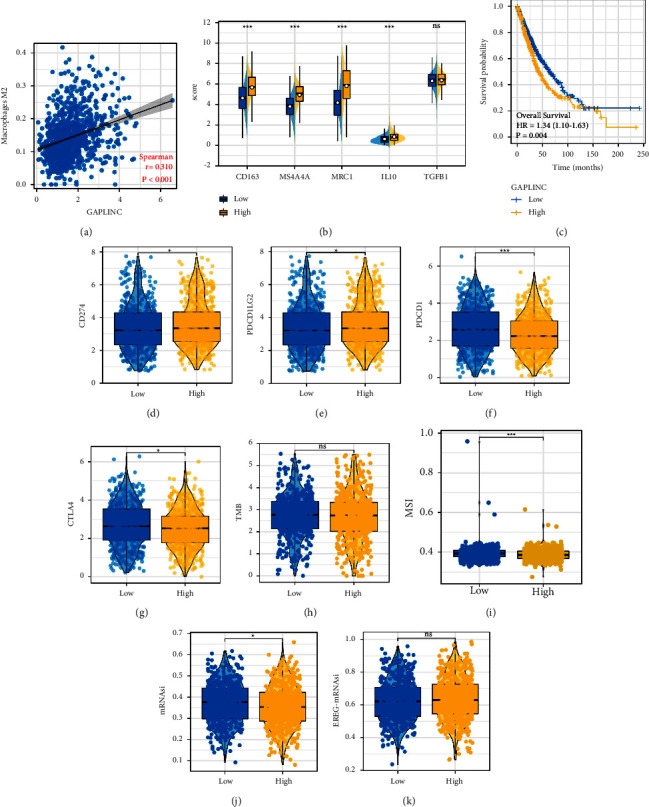
GAPLINC induces M2 macrophages polarization. (a) Correlation between GAPLINC and M2 macrophages based on public data; (b) M2 macrophage-related molecules were highly expressed in the patients with high GAPLINC expression; (c) KM survival curve of patients with high and low GAPLINC expression; (d)–(g) hub immune checkpoints level in patients with high and low GAPLINC expression; (h)–(k) TMB, MSI, mRNAsi, and EREG-mRNAsi difference in patients with high GAPLINC expression.

**Figure 8:**
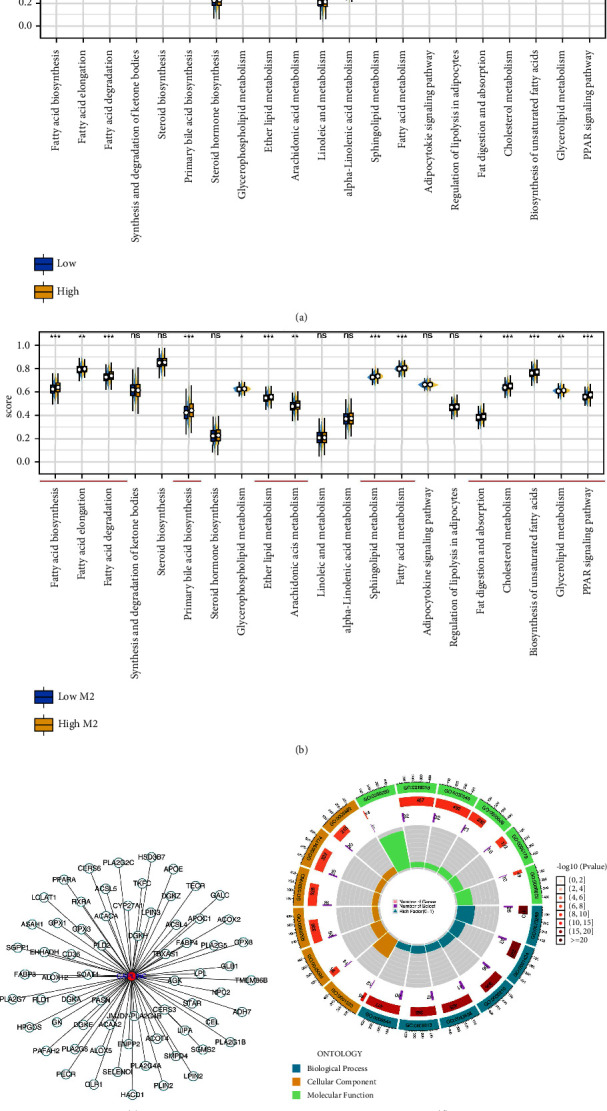
GAPLINC may affect M2 macrophages' polarization through multiple metabolism pathways. (a) Metabolism differences in patients with high and low GAPLINC expression; (b) metabolism differences in patients with high and low M2 macrophage infiltration; (c) GAPLINC affects tumor metabolism and M2 macrophages by regulating specific molecules; (d) GO analysis of GAPLINC.

Based on the public data analysis, we found a positive correlation between GAPLINC and M2 macrophages (Figure 7(a), correlation = 0.310, *P* < 0.001). Also, M2 macrophage-related molecules were overexpressed in the patients with high GAPLINC expression (Figure 7(b)). KM showed that GAPLINC is a risk factor for lung cancer patients (Figure 7(c), HR = 1.34, *P*=0.004). Considering the effect of GAPLINC on lung cancer immunotherapy, we further explored its underlying mechanisms. We observed a higher expression level of PD-1 and PD-L2, while a lower expression level of PD-L1 and CTLA4 was observed in patients with a high GAPLINC level (Figures 7(d)–7(g)). No significant difference was observed in TMB level between patients with high and low GAPLINC expression (Figure 7(h)). Meanwhile, we noticed that the patients with high GAPLINC might have lower MSI and mRNAsi levels (Figures 7(i)–7(j)). Also, no remarkable difference was found in EREG-mRNAsi (Figure 7(k)). Next, we explore the metabolic differences in patients with high and low GAPLINC expression, as well as high and low M2 macrophage infiltration. Results showed that fatty acid biosynthesis, fatty acid elongation, fatty acid degradation, primary bile acid biosynthesis, ether lipid metabolism, arachidonic acid metabolism, sphingolipid metabolism, fatty acid metabolism, fat digestion and absorption, cholesterol metabolism, biosynthesis of unsaturated fatty acids, glycerolipid metabolism, and the PPAR signaling pathway had consistent expression patterns in patients with high GAPLINC and high M2 macrophages (Figures 8(a) and 8(b)). We next identified the molecules that were significantly correlated with GAPLINC and also involved in these metabolism pathways. GAPLINC might affect tumor metabolism and M2 macrophages by regulating these molecules (Figure 8(c)). Finally, GO analysis indicated that GAPLINC was involved in the structural constituent of the skin epidermis (GO: 0030280), receptor-ligand activity (GO: 0048018), signaling receptor activator activity (GO: 0030546), glycosaminoglycan binding (GO: 0005539), hormone activity (GO: 0005179), chemokine activity (GO: 0008009), intermediate filament (GO: 0005882), secretory granule lumen (GO: 0034774), vesicle lumen (GO: 0031983), cytoplasmic vesicle lumen (GO: 0060205), keratin filament (GO: 0045095), cornified envelope (GO: 0001533), epidermis development (GO: 0008544), epidermal cell differentiation (GO: 0009913), skin development (GO: 0043588), keratinocyte differentiation (GO: 0030216), keratinization (GO: 0031424), and cornification (GO: 0070268) (Figure 8(d)).

## 4. Discussion

Lung cancer is still a threatening disease globally. Despite the progress of medical technology, the incidence and death cases of lung cancer still keep rising due to lifestyle and environmental factors [20]. Thus, an in-depth exploration of lung cancer biological mechanisms is important and meaningful.

LncRNAs widely existed in all levels of cells and are famous for their extensive regulation effect [21, 22]. Recently, the association between lncRNAs and metabolism has aroused the interest of researchers. By interacting with RNA, chromatin, and proteins, lncRNAs can correspondingly influence mRNA stability, chromatin structure, and protein function, making them a key factor in tumor metabolism [23]. Previous studies have shown that lncRNAs can regulate HMGCR and LDLR in an SREBP2-dependent manner, further affecting lipid homeostasis [24]. In nasopharyngeal cancer, Zheng et al. indicated that lncRNA TINCR can bind ACLY and prevent its ubiquitin degradation to maintain the total level of intracellular acetyl CoA, further affecting cancer progression and chemoresistance [25]. Also, lncRNAs have been reported to be involved in the regulation of the synthesis of triglycerides, phospholipid metabolism, and so on [26]. Meanwhile, lncRNA has a link with the M2 polarization of macrophages. Cao et al. found that the lncRNA MM2P can affect M2 macrophage polarization by enhancing the phosphorylation of STAT6, thereby promoting cancer progression [9]. Liang et al. revealed that lncRNA RPPH1 could interact with TUBB3, therefore inducing M2 macrophage polarization through an exosome manner [27]. Therefore, identifying the lncRNAs acting as the “bridge” connecting tumor metabolism and M2 macrophages can provide novel insights for future studies.

We first quantified the M2 macrophages through the CIBERSORT algorithm. Results showed that the patients with high M2 macrophage level tended to have worse survival outcomes, and multiple oncogenic pathways were activated. Our results are consistent with previous studies. Chen et al. revealed that miR-19b-3p can promote lung cancer metastasis by inducing M2 macrophage polarization in a Hippo-dependent manner [28]. Lu et al. revealed that Oct4 can contribute to M2 macrophage polarization by increasing the M-CSF level, therefore promoting cancer development [5]. These results reflect the validity of our analysis. The pathways identified can provide direction for the following studies focused on M2 macrophages in lung cancer.

Next, we found that multiple metabolic pathways were associated with M2 macrophages. Some of these have been revealed. Under a normal physiological microenvironment, macrophages can phagocytize and eliminate cell debris and dying cells, which provides nutrition for macrophages [29]. However, in TME, macrophages often undergo metabolic reprogramming and gradually lose the ability to kill tumor cells [30]. Therefore, it is necessary to take into account the complex metabolic regulation of macrophages when studying their interactions with tumor cells. Glucose and glutamine are both sources of fuel for macrophages' tricarboxylic acid (TCA) cycle. M2 macrophages are highly dependent on glutamine influx into the TCA cycle, in contrast to M1 macrophages [31]. Studies showed that macrophage polarization may be affected by the nutrients they consume [32]. Another example is the oxidation of fatty acids, which provides macrophages with a crucial energy source for maturation into M2 macrophages. For this, M2 macrophages internalize triacylglycerol substrate through CD36 and perform lipolysis [33]. The metabolism pathway we identified can improve the metabolism regulatory network of M2 macrophages.

Moreover, we established a prognosis signature based on AC027288.3, AP001189.3, FAM30A, GAPLINC, LINC00578, and LINC01936. The prognosis model showed satisfactory performance in predicting patient survival. Also, patients in different risk groups might respond differentially to immunotherapy and chemotherapy. GAPLINC was selected for further analysis. Previous studies have explored the role of GAPLINC in cancers. Luo et al. found that GAPLINC can facilitate colon cancer progression by affecting the miR-34a/c-MET axis [34]. Also, GAPLINC can increase SNAI2 expression by interacting with PSF and NONO, thereby promoting colon cancer invasion [35]. In gastric cancer, GIAPLINC can modify CD44-dependent cell invasion in cancer cells, which relates to a poor prognosis [36]. Moreover, GAPLINC was found to be involved in the regulation of epithelial-mesenchymal transition, further facilitating liver cancer invasion and migration [37]. Zhao et al. found that the TGF-*β* could facilitate cell invasion and migration in lung cancer by regulating GAPLINC [38]. Nonetheless, few studies concentrated on the interaction between GAPLINC and M2 macrophages. A new understanding of GAPLINC's role in cancer may be provided by our results, especially in lung cancer, making it an underlying biological target.

Several limitations should be discussed. First, as a result of the low proportion of Asian and African populations in the enrolled samples, there may be racial bias in the results. Second, clinical information was incomplete; for example, a large number of patients do not have their M-stage information. Our conclusions would be more credible if all the data were complete and openly accessible.

## Figures and Tables

**Table 1:** The baseline information of the included patients.

Clinical features		Numbers	Percentage (%)
Gender	Female	411	40.1
	Male	615	59.9

Age	≤65	431	42.0
	>65	567	55.3
	Unknown	28	2.7

T stage	T1	286	27.9
	T2	576	56.1
	T3	118	11.5
	T4	43	4.2
	Unknown	3	0.3

N stage	N0	655	63.8
	N1	231	22.5
	N2	115	11.2
	N3	7	0.7
	Unknown	18	1.8

M stage	M0	767	74.8
	M1	32	3.1
	Unknown	227	22.1

Stage	Stage I	524	51.1
	Stage II	287	27.9
	Stage III	170	16.6
	Stage IV	33	3.2
	Unknown	12	1.2

## Data Availability

The data used to support the findings of this study are available from corresponding author upon request.

## Supplementary Materials

Supplementary Materials Figure S1. The flowchart of the whole study. Figure S2. A total of 67 metabolism-related lncRNAs were remarkably correlated with M2 macrophages. Figure S3. Univariate and multivariate analyses of risk scores. Figure S4. Biological enrichment of model lncRNAs. (A) GSEA analysis of AC027288.3 based on the hallmark gene set; (B) GSEA analysis of AP001189.3 based on the Hallmark gene set; (C) GSEA analysis of FAM30A based on the hallmark gene set; (D) GSEA analysis of GAPLINC based on the hallmark gene set; (E) GSEA analysis of LINC00578 based on the Hallmark gene set; (F) GSEA analysis of LINC01936 based on hallmark gene set. Figure S5. Correlation between risk score and other macrophage subtypes. (A) Correlation between risk score and M0 macrophage; (B) correlation between risk score and M1 macrophage.
