# Supplementary material for: Exploration of the Long Noncoding RNAs Involved in the Crosstalk between M2 Macrophages and Tumor Metabolism in Lung Cancer
Source: Genet Res (Camb). 2023 Jan 25;2023:4512820. doi: 10.1155/2023/4512820 (PMC9891836; doi:10.1155/2023/4512820)
Supplement: Supplementary Materials — Figure S1. The flowchart of the whole study. Figure S2. A total of 67 metabolism-related lncRNAs were remarkably correlated with M2 macrophages. Figure S3. Univariate and multivariate analyses of risk scores. Figure S4. Biological enrichment of model lncRNAs. (A) GSEA analysis of AC027288.3 based on the hallmark gene set; (B) GSEA analysis of AP001189.3 based on the Hallmark gene set; (C) GSEA analysis of FAM30A based on the hallmark gene set; (D) GSEA analysis of GAPLINC based on the hallmark gene set; (E) GSEA analysis of LINC00578 based on the Hallmark gene set; (F) GSEA analysis of LINC01936 based on hallmark gene set. Figure S5. Correlation between risk score and other macrophage subtypes. (A) Correlation between risk score and M0 macrophage; (B) correlation between risk score and M1 macrophage. [file 4512820.f1.zip › Figure S3.pdf]

# Figure S3

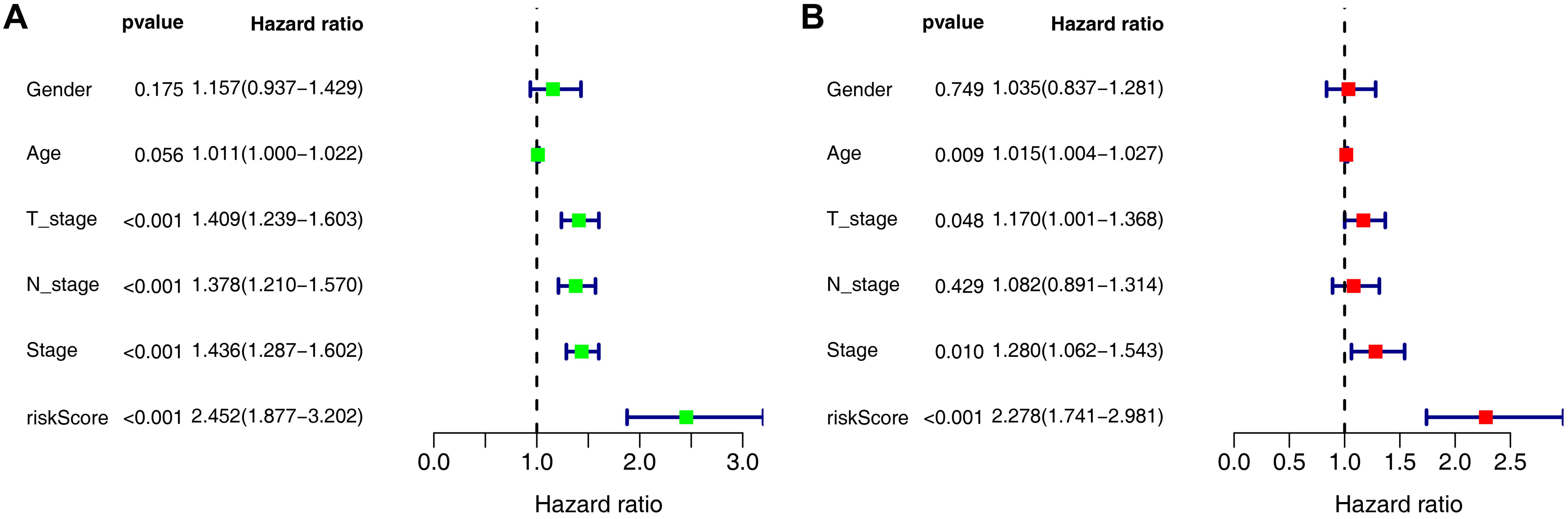

A
pvalue
Hazard ratio
Gender 0.175 1.157(0.937−1.429)
Age 0.056 1.011(1.000−1.022)
T_stage <0.001 1.409(1.239−1.603)
N_stage <0.001 1.378(1.210−1.570)
Stage <0.001 1.436(1.287−1.602)
riskScore <0.001 2.452(1.877−3.202)
0.0
1.0
2.0
3.0
Hazard ratio
B
pvalue
Hazard ratio
Gender 0.749 1.035(0.837−1.281)
Age 0.009 1.015(1.004−1.027)
T_stage 0.048 1.170(1.001−1.368)
N_stage 0.429 1.082(0.891−1.314)
Stage 0.010 1.280(1.062−1.543)
riskScore <0.001 2.278(1.741−2.981)
0.0
0.5
1.0
1.5
2.0
2.5
Hazard ratio
